# A quadratically constrained mixed-integer non-linear programming model for multiple sink distributions

**DOI:** 10.1016/j.heliyon.2024.e38528

**Published:** 2024-10-01

**Authors:** Bernard Atta Adjei, Charles Sebil, Dominic Otoo, Joseph Ackora-Prah

**Affiliations:** a Department of Mathematics and Statistics, University of Energy and Natural Resources, Sunyani, Box 214, Bono Ahafo, Ghana; b Department of Mathematics, Kwame Nkrumah University of Science and Technology, Kumasi, Box Up 1279, Ashanti, Ghana

**Keywords:** 90C30, 90C11, 90C06, 90B20, 90B06, Quadratically constrained mixed-integer nonlinear programming, Vehicle routing problem, Intercity distribution

## Abstract

Rising traffic congestion and fuel costs pose significant challenges for supply chains with numerous retailers. This paper addresses these challenges by optimizing transportation routes for processed tomatoes within a long-haul and intercity distribution network. We use the heterogeneous capacitated vehicle routing problem framework to create a new quadratically constrained mixed-integer non-linear programming model that aims to meet demand at multiple destinations while minimizing transportation costs. Our model incorporates real-time data and route optimization strategies that consider traffic conditions based on freight time and route diversions for expedited deliveries. It aims to devise an optimal transportation schedule that minimizes fuel, operational, and maintenance costs while ensuring efficient delivery of tomato paste. By applying this model to a real-world case study, we estimate a significant 27.59% reduction in transportation costs, dropping them from GH¢20,270 ($1,638.91) to GH¢14,676 ($1,186.61) on average. This highlights the effectiveness of our strategy in lowering costs while maintaining smooth and efficient deliveries.

## 1 Background

The Weddi Africa Tomato Processing and Agro Farm is a $16 million tomato processing factory operated entirely by a Ghanaian factory in Domfete, Bono Region [6]. After the tomato manufacturing process at the factory, the next critical step is ensuring effective transportation of their products to the distribution centers (warehouse), which is situated in the heart of one of the most densely populated metropolitan areas in Ghana (Kumasi). In any well-functioning supply chain management system, distribution centers hold significant importance [1]. According to research, the presence of these distribution centers highlights the essential role of transportation management as a logistical concern for organizations [9], [19]. This is because transportation forms a considerable portion of their overall logistical expenses.

The transportation of processed tomatoes incurs high costs due to the location of the distribution centers within a congested traffic zone, and the dispersed retailers pose transportation challenges that require an optimal approach, especially when distributing to multiple destinations with varying demand levels for five different products. The traffic congestion impacts delivery times, leading to potential delays that can compromise efficiency. Fuel costs are another critical factor, as they constitute a substantial portion of the total transportation expenses. Addressing these challenges with an optimized model is economically significant, potentially reducing costs and improving efficiency in the supply chain. Furthermore, the heterogeneous nature of the vehicle fleet, with varying capacities and operational costs, adds complexity to route optimization. Ensuring that each vehicle is utilized efficiently while minimizing the number of trips and maintaining delivery schedules is a significant logistical challenge.

The objective of this paper is therefore to develop a mathematical model that minimizes the costs associated with transporting “Sweet Mama Tomatoes Mix,” a branded tomato paste produced by the factory, from the distribution centers to various retailers. This optimal process is achieved while ensuring that the demand for tomato paste at each destination is adequately met. Most transportation problems studied emerge from the fact that vehicle routing problems pose complex optimization challenges with widespread applications in various sectors, including manufacturing, logistics, transportation, and communication. They are ranked among the most extensively studied combinatorial optimization problems [14].

In many studies addressing transportation issues, researchers often focus on moving a single type of goods using a uniform fleet of vehicles, overlooking various real-world factors related to trucks. However, most existing transportation models fall short of representing our specific problem. This is because the amount of goods transported cannot exceed the capacity of the carrier, leading to multiple trips. The cost of transportation is also somehow tied to the optimal size of the vehicle, as pointed out by previous research [23]. Once again, previous research underlines the importance of optimizing vehicle usage within the transportation process [2], [7], [22], [28]. To address these limitations, scholars have turned to optimization techniques such as nonlinear programming (NLP) and mixed-integer nonlinear programming (MINLP). They've directed their efforts towards rectifying the shortcomings in the current transportation models. Specifically, in the context of the cVRP, the challenge involves delivering goods to various locations using vehicles with limited carrying capacity.

In response to these challenges, this paper looks at whether mathematical programming models can effectively optimize transportation routes for processed tomatoes, leading to efficient operations and significant cost savings by incorporating real-time traffic data and addressing the heterogeneous nature of the vehicle fleet. By incorporating real-time traffic data, the model aims to minimize fuel, operational, and maintenance costs while ensuring timely and efficient deliveries. The application of this model to a real-world case study demonstrates its potential to significantly reduce transportation costs and improve the overall efficiency of the distribution network. Furthermore, this study also extends its tentacle to the optimal loading of goods using the model result and PackVol software to ensure smooth deliveries.

## 2 Related works

There is resilience in transportation systems, which has been extensively researched over the last decade [34]. The purpose of this section is to provide a synthesis of the most recent literature on optimal transportation, with a focus on concepts and methodologies.

A study provided an overview of the most relevant research projects undertaken to address natural gas transportation issues via pipeline systems [29]. Their work aimed to present a thorough examination of the efforts made to optimize natural gas transmission lines. It was concluded that one of the major challenges to efficiently exploiting natural gas supplies stems from the limitations of optimization techniques, which have already been developed in theory but are less applicable in practice due to significantly strong assumptions. According to other works, most oil companies cannot be productive and competitive and thus will not survive if supply chain management concepts are not considered [17]. The authors conducted a thorough examination of mathematical programming models used in the supply chain management of oil companies. It was concluded that efficient algorithms for solving these complex large-scale models as translations of realistic real-size problems had been developed. Complex mathematical models are widely used in our modern computer age to solve a wide range of difficult problems in science and engineering [10].

[15] presented a robust approach to designing and restructuring logistics networks for retailers. By integrating strategic decisions on the number and types of DCs with tactical planning considerations, the proposed mixed-integer programming (MIP) model and iterated local search algorithm offer a comprehensive solution to the distribution network design problem. The findings highlighted the substantial improvement potential in logistics cost efficiency and resilience that can be achieved through strategic network restructuring.

Existing routing algorithms are mostly based on mathematical programming, which requires a lot of computation time in city-sized transportation networks [16]. The authors propose a novel deep reinforcement learning-based neural combinatorial optimization strategy for developing routes in the shortest amount of time. Their simulation results show that the proposed strategy outperforms conventional strategies in both static and dynamic logistic systems with limited computation time. Another study also used the LP model to determine the maximum flow of vehicles and commodities that can be accommodated on different transportation modes within multi-modal transportation systems (MMTS) over a given time period [4]. Multi-modal planning takes into account various modes (walking, cycling, driving, public transportation, and so on) as well as connections between modes [20]. Their model was able to determine how commodities are moved between different origin-destination pairs and facilitate commodity transfers across different modes. The authors concluded that their analysis indicates that the proposed capacity assessment techniques are valuable and may aid transportation planners in the development of better MMTS.

[30] presented a comprehensive approach to optimizing the supply chain for perishable products, addressing key challenges through integrated location, inventory, and routing strategies. The location-inventory problem (LIP) formulated as a (MINLP) model of their study utilizes CPLEX, an optimization solver, to find solutions, and for the location-inventory-routing problem (LIRP) formulated as a mixed integer linear programming (MILP) model, a two-stage heuristic algorithm based on simulated annealing was designed to address the complexity of the problem. The findings illustrated the substantial cost-saving potential of direct shipments from suppliers to retailers and provided a road map for future enhancements in the perishable product supply chain, particularly in the context of e-retail growth.

A study envisioned a supply chain network that included local and global medical relief item suppliers, regional and central distribution centers, and numerous customer demand points. A capacitated multi-stage operations scheduling problem with renewable and non-renewable resources was presented in an emergency supply chain [12]. For large-scale problems, the authors used the particle swarm optimization algorithm. Managers can plan and decide whether to increase their pre-planned inventory of standard kits and medical relief items based on their presented results. Another study also highlighted the current state-of-the-art of various advanced tools used to reduce fruit and vegetable quality loss during packaging, storage, and transportation cold chain operations [26]. Other methods, such as a mathematical optimization model, were used in their work. The authors concluded that future research should focus on integrating the internet of things and digital twins for multiple shipments in order to improve real-time monitoring of cold chain environmental conditions and eventually optimize post-harvest supply chains.

[33] developed a comprehensive and robust approach to humanitarian relief network design, integrating key operational functions under uncertainty. The distributionally robust MILP model and the enhanced branch-and-cut algorithm demonstrated strong performance in providing reliable and efficient solutions. The findings emphasize the importance of an integrated approach in disaster response operations and offer valuable insights for improving the effectiveness of humanitarian relief efforts.

A Mixed-Integer Quadratic Programming (MIQP) model was created to optimally distribute goods to 105 distributors across Ghana from two factories [3]. The developed model and analysis show that having multiple vehicles in a fleet for long hauls of goods results in an optimal minimum cost when compared to a single-vehicle fleet. Again, the author claimed that a single-vehicle fleet with loading capacity within the mean value of all individual demands resulted in a cost that was close to the optimal minimum. Another model was created using robust fuzzy programming to investigate the effects of uncertainty parameters such as customer demand, fraction of returned products, transportation costs, raw material prices, and shortage costs [13]. Because their developed model was NP-hard, they proposed a novel whale optimization algorithm aimed at minimizing network total costs through the use of a modified priority-based encoding procedure. The authors concluded that their solution is an indication of the applied algorithm's high-quality performance in finding a near-optimal solution in a reasonable computational time.

[31] presented a comprehensive approach to designing multi-class hazmat distribution networks by integrating location, inventory, and routing decisions while considering superimposed risks. Their findings underscored the importance of trade-offs between risk minimization, risk equilibration, service level, and economic viability in hazmat logistics. The proposed NSGA-II-CD algorithm demonstrated effective problem-solving capabilities, offering practical implications for enhancing hazmat logistics management in metropolitan areas.

[32] presented the integration of location, routing, and inventory decisions in a multi-period location inventory routing problem (LIRP) with fuel consumption and an effective approach to supply chain optimization. The two-stage hybrid meta-heuristic algorithm demonstrated robust performance, significantly reducing total costs and offering practical solutions for complex supply chain problems. The study's findings underscored the importance of considering both transportation and inventory costs in supply chain management and provided actionable insights for enhancing operational efficiency.

Traditional transportation models like the North-West Corner, Least Cost, and Vogel's Approximation Methods often focus on the movement of a single type of goods using a homogeneous fleet of vehicles, overlooking the complexities introduced by real-world constraints such as vehicle heterogeneity and traffic conditions. Linear and nonlinear existing models inadequately omit the set, which includes vehicles of varying capacities, and parameters, which include product specifications such as weight and volume, as well as vehicle dimension. This limitation is critical, as optimizing the use of a mixed fleet can significantly impact overall transportation efficiency. Many studies do not also incorporate real-time data, such as average route speed, for route optimization. This is essential for adjusting routes based on current traffic conditions, which can affect delivery times and costs.

## 3 Problem definition

**Figure 1:**
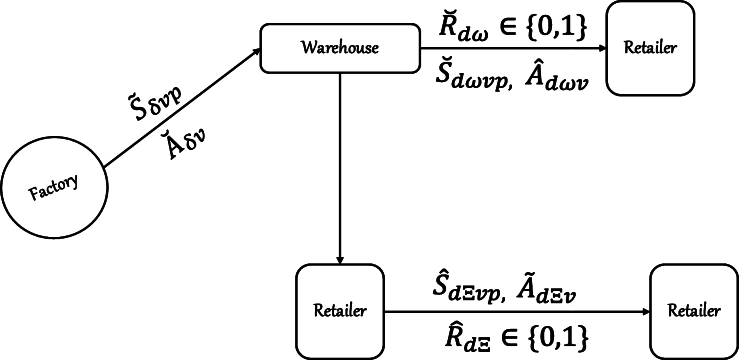
The underlying transportation networks.

The transportation of Sweet Mama Tomato Mix poses significant logistical challenges due to its varying demand at multiple heavily congested traffic destinations and the need for cost efficiency. Rising fuel costs, traffic congestion, and the complexity of managing a heterogeneous vehicle fleet further worsen these challenges. Looking at the transportation structure, the problem is designed in two stages: long-haul transportation and intercity distribution. The problem at hand revolves around deterministic planning, wherein the demand is known in advance. It is crucial to acknowledge that significant decisions are influenced by factors like traffic conditions, distances between different distributors and retailers, and travel times. Fig. 1 shows the underlying transportation networks.

The transportation problem in this paper was solved by modeling a heterogeneous capacitated vehicle routing problem (hc-VRP). This approach takes into account a diverse group of vehicles, each with its own unique capacity and operating costs. Factors such as vehicle axle weight and product weight are considered in conjunction with truck and product specification data. The transportation itself is represented using a commodity flow formulation. The total quantity of goods is quantified and divided based on the mode of transportation, the type of commodity, and the nature of the cargo. This approach allows for the distribution of a larger number of requested products across multiple time frames in smaller quantities.

The distribution of goods to various retailers is carried out over a span of six working days, from Monday to Saturday. Within each day, distribution activities are divided into three distinct time frames: morning, afternoon, and evening. Each of these time frames experiences varying traffic conditions. Moreover, there can be restrictions on the number of retailers that can be serviced on any given day. With the presence of numerous routes characterized by varying traffic conditions throughout the week, the model is formulated to determine a feasible solution that achieves the following objectives:

• Employing the best delivery time for each day.
• Selecting the optimal route for each working day.
• Meeting all demands within the course of a week, either at once or in batches.

### 3.1 The proposed mathematical model

**Figure 2:**
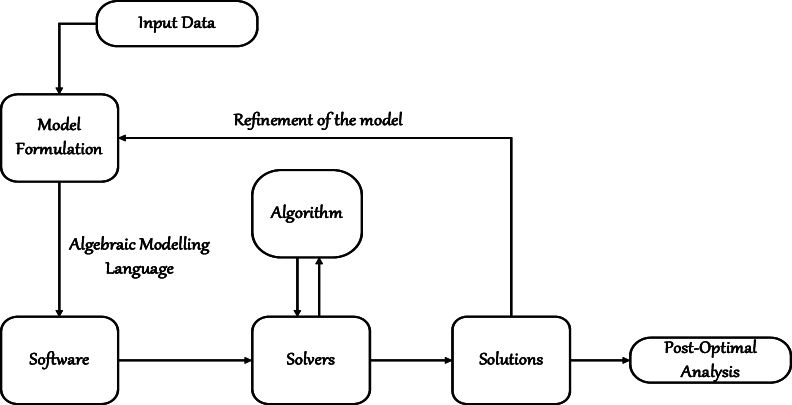
Schematic representation of the proposed approach.

Fig. 2 shows a schematic of the proposed approach that illustrates and clarifies the methodology, enhancing the understanding of the underlying processes and the overall strategy.

**Indexes**

The index notation is used to specify the items of an array. The location of elements in a sequence is represented numerically by indexes. As demonstrated in Table 1, Table 2, a sequence can be a list, a character string, or any other random succession of data.

**Table 1:** List of all single indexes.

Index	Description
*i* = 1,2,⋯,*I*:	location of all factories.
*p* = 1,2,⋯,*P*:	set of available finished product.
*w* = 1,2,⋯,*W*:	set of warehouses/distribution centers
*s* = 1,2,⋯,*S*:	location of all retailers.
*v* = 1,2,⋯,*V*:	set of all vehicles.
*d* = 1,2,⋯,*D*:	set of days.
*t* = 1,2,⋯,*T*:	set of distribution time.

**Table 2:** List of all multiple indexes.

Index	Description
*δ*:	from factory (*i*) transporting to warehouse (*w*).
*γ*:	from retailer (*s*) transporting to retailer (*m*).
*κ*:	from warehouse (*w*) transporting to retailer (*s*).
*ω*:	from warehouse (*w*) transporting to retailer (*s*) at time (*t*).
*ξ*:	from retailer (*s*) transporting to retailer (*m*) at time (*t*).

The sets listed below are connected to other sets. This kind of index is used to divide a certain collection. To put it another way, one set is a subset (⊆) of another.

**Variables**

$M_{dwp}$: the total number of each product *p* transported from the warehouse *w* at each given day *d*.

${\overset{˜}{S}}_{\delta vp}$: the number of products *p* shipped from factory *i* to warehouse *w* using vehicle *v*.

${\overset{˘}{S}}_{d\omega vp}$: the number of products *p* shipped from warehouse *w* to retailers *s* at a given day *d* and time *t* using vehicle *v*.

${\overset{ˇ}{S}}_{d\xi vp}$: the number of products *p* shipped during a continuous transportation from retailer *s* to retailer *m* at a given day *d* and time *t* using vehicle *v*.

${\overset{˘}{A}}_{\delta v}$: the number of vehicles *v* needed to ship from factory *f* to warehouse *w*.

${\overset{ˇ}{A}}_{d\omega v}$: the number of vehicles *v* needed to ship from warehouse *w* to retailer *s* in a given day *d* and time *t*.

${\overset{˜}{A}}_{d\xi v}$: the number of vehicles *v* needed to perform a continuous shipment from retailer *s* to retailer *m* in a given day *d* and time *t*.

${\overset{˘}{R}}_{d\omega }$: a binary variable that activates the shipment of product from warehouse *w* to retailer *s* in a given day *d* and time *t*.

${\overset{ˆ}{R}}_{d\xi }$: a binary variable that activates the shipment of product from retailer *s* to retailer *m* in a given day *d* and time *t*.

$\tau_{dt}$: measures the traveling time in a given day *d* and time *t*.

$y_{v}$: the number of trips each vehicle *v* can make in a day.

**Input data/Parameter**

${\overset{˘}{f}}_{ip}$: the number of products *p* ready to be transported at factory *i*.

${\overset{ˇ}{v}}_{v}$: cargo capacity of vehicle *v*.

${\overset{ˆ}{m}}_{v}$: cost of maintenance of vehicle *v*.

${\overset{˘}{m}}_{v}$: weight of vehicle *v*.

$\overset{ˇ}{m}$: fuel price per liter.

${\overset{ˆ}{e}}_{p}$: dimensional size of finished product in box *p*.

${\overset{˘}{e}}_{p}$: weight of finished product in box *p*.

${\overset{˘}{d}}_{sp}$: the number of products *p* demanded by individual retailers *s*.

${\overset{ˆ}{r}}_{iw}$: distance from factory *i* to warehouse *w*.

${\overset{ˇ}{r}}_{ws}$: distance from warehouse *w* to retailer *s*.

${\overset{˘}{r}}_{sm}$: distance from retailer *s* to retailer *m*.

${\overset{ˆ}{j}}_{\delta }$: the average traveling speed from factory *i* to warehouse *w*.

${\overset{ˇ}{j}}_{d\omega }$: the average traveling speed from warehouse *w* to retailer *s* in a given day *d* and time *t*.

${\overset{˘}{j}}_{d\xi }$: the average traveling speed from retailer *s* to retailer *m* in a given day *d* and time *t*.

${\overset{ˆ}{t}}_{\delta }$: the average traveling time from factory *i* to warehouse *w*.

${\overset{ˇ}{t}}_{d\omega }$: the average traveling time from warehouse *w* to retailer *s* in a given day *d* and time *t*.

${\overset{˘}{t}}_{d\xi }$: the average traveling time from retailer *s* to retailer *m* in a given day *d* and time *t*.

${\overset{ˆ}{l}}_{\delta v}$: the average quantity of fuel in liter from factory *i* to warehouse *w* at time *t* using vehicle *v*.

${\overset{ˇ}{l}}_{d\omega v}$: the average quantity of fuel in liter from warehouse *w* to retailer *s* in a given day *d* and time *t* using vehicle *v*.

${\overset{˘}{l}}_{d\xi v}$: the average quantity of fuel in liter from retailer *s* to retailer *s* in a given day *d* and time *t* using vehicle *v*.

${\overset{ˆ}{n}}_{\delta v}$: the estimated vehicle operational cost from factory *i* to warehouse *w* using vehicle *v*.

${\overset{ˇ}{n}}_{d\omega v}$: the estimated vehicle operational cost from warehouse *w* to retailer *s* in a given day *d* and time *t* using vehicle *v*.

${\overset{˘}{n}}_{d\xi v}$: the estimated vehicle operational cost from retailer *s* to retailer *m* in a given day *d* and time *t* using vehicle *v*.

*ψ*: the dimensional error of the cargo loading.

*γ*: capacity of warehouses.

$\varepsilon_{v}$: fuel consumption per hour for vehicle *v*.

**Objective Function**

The objective is to minimize the objective function Equation (1), which represents the total vehicle operational costs. It's worth noting that Ω is indexed on (w,s,t) respectively for warehouse, retailer, and time, and Ξ is indexed on (s,m,t) respectively for retailer to retailer and time. It's also worth noting that *δ* is indexed in two sets: *i* for factories and *w* for warehouses. *γ* is indexed in two sets: *s* for retailer and *m* for another retailer. *κ* is indexed on two sets: *w* for warehouse and *s* for retailer.

(1) $$F({\overset{˘}{A}}_{\delta v},{\overset{ˇ}{A}}_{d\omega v},{\overset{˜}{A}}_{d\xi v})=\sum_{\delta v}^{\Delta V}{\overset{ˆ}{n}}_{\delta v}{\overset{˘}{A}}_{\delta v}+\sum_{d\omega v}^{D\Omega V}{\overset{ˇ}{n}}_{d\omega }{\overset{ˇ}{A}}_{d\omega v}+\sum_{d\xi v}^{D\Xi V}{\overset{˘}{n}}_{d\xi }{\overset{˜}{A}}_{d\xi v}\;$$

**Constraints**

Equations (2) - (36) provide the constraints for this model.

(2) $$\sum_{vw}^{VW}{\overset{˜}{S}}_{\delta vp}\leq {\overset{˘}{f}}_{ip},\;\;\text{for all }(i,p)\;$$

(3) $$\sum_{vw}^{VW}{\overset{˜}{S}}_{\delta vp}\geq \sum_{ds}^{DS}{\overset{˘}{d}}_{sp},\;\;\text{for all }(i,p)\;$$

(4) $$\sum_{d}^{D}M_{dwp}=\sum_{v}^{V}{\overset{˜}{S}}_{\delta vp},\;\;\text{for all }(\delta ,p)\;$$

(5) $$\sum_{tsv}^{TSV}{\overset{˘}{S}}_{d\omega vp}\leq M_{dwp},\;\;\text{for all }(d,w,p)\;$$

(6) $$\sum_{\omega v}^{\Omega V}{\overset{˘}{S}}_{d\omega vp}+\sum_{\xi v}^{\Xi V}{\overset{ˇ}{S}}_{d,\xi^{⁎}vp}={\overset{˘}{d}}_{kp}+\sum_{\xi ,v}^{\Xi v}{\overset{ˇ}{S}}_{d\xi vp},\;\;\text{for all }(m,p)\;$$

(7) $$\sum_{p}^{P}{\overset{ˆ}{e}}_{p}{\overset{˜}{S}}_{\delta vp}\leq {\overset{ˇ}{v}}_{v}{\overset{˘}{A}}_{\delta v}\psi ,\;\;\text{for all }(\delta ,v)\;$$

(8) $$\sum_{p}^{P}{\overset{ˆ}{e}}_{p}{\overset{˘}{S}}_{d\omega vp}\leq {\overset{ˇ}{v}}_{v}{\overset{ˇ}{A}}_{d\omega v}{\overset{˘}{R}}_{d\omega }\psi ,\;\;\text{for all }(d,\Omega ,v)\;$$

(9) $$\sum_{p}^{P}{\overset{ˆ}{e}}_{p}{\overset{ˇ}{S}}_{d\xi vp}\leq {\overset{ˇ}{v}}_{v}{\overset{˜}{A}}_{d\xi v}{\overset{ˆ}{R}}_{d\xi },\;\;\text{for all }(d,\xi ,v)\;$$

(10) $$\sum_{p}^{P}{\overset{˘}{e}}_{p}{\overset{˜}{S}}_{\delta vp}\leq {\overset{˘}{m}}_{v}{\overset{˘}{A}}_{\delta v},\;\;\text{for all }(\delta ,v)\;$$

(11) $$\sum_{p}^{P}{\overset{˘}{e}}_{p}{\overset{˘}{S}}_{d\omega vp}\leq {\overset{˘}{m}}_{v}{\overset{ˇ}{A}}_{d\omega v}{\overset{˘}{R}}_{d\omega },\;\;\text{for all }(d,\omega ,v)\;$$

(12) $$\sum_{p}^{P}{\overset{˘}{e}}_{p}{\overset{ˇ}{S}}_{d\xi vp}\leq {\overset{˘}{m}}_{v}{\overset{˜}{A}}_{d\xi v}{\overset{ˆ}{R}}_{d\xi },\;\;\text{for all }(d,\xi ,v)\;$$

(13) $$\sum_{\omega }^{\Omega }{\overset{ˇ}{A}}_{d\omega v}-\sum_{\xi }^{\Xi }{\overset{˜}{A}}_{d\xi v}\leq y_{v},\;\;\text{for all }(d,v)\;$$

(14) $$\sum_{\xi }^{Xi}{\overset{˜}{A}}_{d\xi v}+\sum_{\xi }{\overset{˜}{A}}_{d\xi^{⁎}v}\leq y_{v},\;\;\text{for all }(d,v)\;$$

(15) $$\tau_{dt}=\sum_{\omega }^{\Omega }{\overset{ˇ}{t}}_{d\omega }{\overset{˘}{R}}_{d\omega }+\sum_{\xi }^{\Xi }{\overset{˘}{t}}_{d\xi }{\overset{ˆ}{R}}_{d\xi }\;\;\text{for all }(d,t)\;$$

(16) $$\sum_{\omega }^{\Omega }{\overset{˘}{S}}_{d\omega vp}+\sum_{\xi }^{\Xi }{\overset{ˇ}{S}}_{d\xi vp}\geq \sum_{\xi^{⁎}}^{\Xi }{\overset{ˇ}{S}}_{d\xi^{⁎}vp},\;\;\text{for all }(d,v,m,p,t)\;$$

(17) $$\sum_{\omega }^{\Omega }{\overset{ˇ}{A}}_{d\omega v}{\overset{˘}{R}}_{d\omega }+\sum_{\xi }^{\Xi }{\overset{˜}{A}}_{d\xi v}{\overset{ˆ}{R}}_{d\xi }\geq \sum_{\xi^{⁎}}^{\Xi }{\overset{˜}{A}}_{d\xi^{⁎}v},\;\;\text{for all }(d,v,m,p,t)\;$$

(18) $$\sum_{vip}^{VIP}{\overset{˜}{S}}_{\delta vp}\leq \gamma ,\;\;\text{for all }(w)\;$$

**Variable Condition**

Equations (19) and (20) define variables as non-negative boolean.

(19) $${\overset{˘}{R}}_{d\delta }\in \{0,1\},\;\;\;\text{for all }(d,\delta )\;$$

(20) $${\overset{ˆ}{R}}_{d\xi }\in \{0,1\},\;\;\;\text{for all }(d,\xi )\;$$

Equations (21) to (27) define variables as non-negative integers.

(21) $$M_{dwp}\in Z\;\;\geq 0,\;\;\;\;\text{for all }(d,w,p)\;$$

(22) $${\overset{˜}{S}}_{\delta vp}\in Z\;\;\geq 0,\;\;\;\;\text{for all }(\delta ,v,p)\;$$

(23) $${\overset{˘}{S}}_{d\omega vp}\in Z\;\;\geq 0,\;\;\;\;\text{for all }(d,\Omega ,v,p)\;$$

(24) $${\overset{ˇ}{S}}_{d\xi vp}\in Z\;\;\geq 0,\;\;\;\;\text{for all }(d,\xi ,v,p)\;$$

(25) $${\overset{˘}{A}}_{\delta v}\in Z\;\;\geq 0,\;\;\;\;\text{for all }(\delta ,v)\;$$

(26) $${\overset{ˇ}{A}}_{d\omega v}\in Z\;\;\geq 0,\;\;\;\;\text{for all }(d,\omega ,v)\;$$

(27) $${\overset{˜}{A}}_{d\xi v}\in Z\;\;\geq 0,\;\;\;\;\text{for all }(d,\Xi ,v)\;$$

**Parameter definitions**

Equations (28) to (36) compute the transportation cost.

(28) $${\overset{ˆ}{t}}_{\delta }={\overset{ˆ}{r}}_{iw}/{\overset{ˆ}{j}}_{\delta },\;\;\;\;\text{for all }(\delta )\;$$

(29) $${\overset{ˇ}{t}}_{d\omega }={\overset{ˇ}{r}}_{ws}/{\overset{ˇ}{j}}_{d\omega },\;\;\;\;\text{for all }(d,\omega )\;$$

(30) $${\overset{˘}{t}}_{d\xi }={\overset{˘}{r}}_{sm}/{\overset{˘}{j}}_{d\xi },\;\;\;\;\text{for all }(d,\Xi )\;$$

(31) $${\overset{ˆ}{l}}_{\delta v}={\overset{ˆ}{t}}_{\delta }\times \varepsilon_{v},\;\;\;\;\text{for all }(\delta ,v)\;$$

(32) $${\overset{ˇ}{l}}_{d\omega v}={\overset{ˇ}{t}}_{d\omega }\times \varepsilon_{v},\;\;\;\;\text{for all }(d,\omega ,v)\;$$

(33) $${\overset{˘}{l}}_{d\xi v}={\overset{˘}{t}}_{d\xi }\times \varepsilon_{v},\;\;\;\;\text{for all }(d,\Xi ,v)\;$$

(34) $${\overset{ˆ}{n}}_{\delta }={\overset{ˆ}{m}}_{v}{\overset{ˆ}{r}}_{iw}+\overset{ˇ}{m}{\overset{ˆ}{l}}_{\delta v},\;\;\;\;\text{for all }(\delta ,v)\;$$

(35) $${\overset{ˇ}{n}}_{d\omega }={\overset{ˆ}{m}}_{v}{\overset{ˇ}{r}}_{ws}+\overset{ˇ}{m}{\overset{ˇ}{l}}_{d\omega v},\;\;\;\;\text{for all }(d,\Omega ,v)\;$$

(36) $${\overset{˘}{n}}_{d\xi }={\overset{ˆ}{m}}_{v}\overset{˘}{sm}+\overset{ˇ}{m}{\overset{˘}{l}}_{d\xi v},\;\;\;\;\text{for all }(d,\xi ,v)\;$$

Equation (2) serves as a validation mechanism to ensure that the quantity of finished products shipped from the factory to the warehouse is within the limits of the factory's production capacity. Equation (3) is an important constraint that ensures that the quantity of products shipped from the factory to the warehouse is enough to meet the demand of retailers. Equation (4) is formulated as a summation over all vehicles and all products, which means that it takes into account the contribution of each vehicle and product to the warehouse inventory. Equation (5) limits the quantity of the first shipment from the warehouse to the individual retailer to the quantity of each product currently in inventory. Equation (6) is an important balancing constraint in the supply chain model. So here, Ω is indexed on (w,k,t), where *k* denotes a retailer; $\Xi^{⁎}$ is also indexed on (s,k,i); and Ξ is indexed on (k,m,t), where *m* denotes another retailer. Using retailer *k* as a point of reference, the shipment from the warehouse *w* to retailer *k* and from a retailer say *s* to retailer *k* equals the demand *d* of retailer *k* and the shipment leaving retailer *k* for another retailer say *m*.

Equations (7) and (10) ensured that the number of vehicles needed to transport the products from the factory to the warehouse are calculated correctly. Additionally, they checked that the vehicle's capacity and weight were within the limits specified. Equations (8), (9), (11) and (12) served the same purpose as Equations (7) and (10) for shipment from warehouse to retailers and between retailers, respectively. Equations (13) and (14) ensured that the intercity distribution was carried out effectively without overburdening the available vehicles. The constraints put an upper bound on the number of trips each vehicle can make, thereby ensuring that each vehicle is optimally utilized. Equation (15) is an important constraint in the logistics optimization problem as it calculates and limits the total travel time in intercity distribution. The total travel time is calculated by summing up the travel time from the origin to the destination. Equations (16) and (17) respectively controls the quantity of each product during intercity shipments and the number of vehicles used. Equation (18) ensures that the quantity of goods transported to each warehouse does not exceed the capacity of the warehouse.

### 3.2 Description of the model

The formulated model is quadratically constrained mixed-integer non-linear programming (QCMINLP), which will be computed using the GuRoBi solver package in AMPL. GuRoBi solver was used due to its ability to handle quadratic constrained non-linear programming models [24]. In order to minimize the number of trucks in the system, according to the objective function Equation (1), the cost is dependent on the number of trucks used, that is, [${\overset{˘}{A}}_{\Delta v}$, ${\overset{ˇ}{A}}_{d\Omega v}$, ${\overset{˜}{A}}_{d\Xi v}$]. Thus, the lower the number of trucks, the lower the transportation cost.

To solve the model presented in this paper, the GuRoBi solver package in AMPL was employed. GuRoBi is chosen for its robustness in handling complex quadratic constraints and non-linear programming models effectively. This solver is well-suited for large-scale optimization problems like the hc-VRP, where multiple variables and constraints need to be managed simultaneously. The GuRoBi solver utilizes advanced mathematical algorithms and heuristic methods to explore feasible solutions efficiently. It performs branch-and-bound and cutting-plane techniques to iteratively refine the solution space, ensuring that the optimal or near-optimal solution is found within a reasonable computational time.

### 3.3 Validation of the methodology

To validate the methodology and modes with existing work, we compared our methodology with related studies. This model of the study addresses the hc-VRP by considering a diverse group of vehicles with different capacities and operating costs. Previous studies on vehicle routing, such as those by [8] and [18], [22], have explored similar problems but often under more simplified assumptions. The model incorporates real-time data and route optimization strategies, which aligns with recent advancements in logistics optimization using dynamic data. For instance, [27] employed reinforcement learning for real-time route optimization in logistics, highlighting the growing trend towards integrating real-time data in optimization models. The approach used in this study is a robust method for handling complex constraints and non-linearity in optimization problems. Similar techniques have been used in various studies for supply chain and logistics optimization, as noted by [11] in multi-objective supply chain scheduling problems. The use of the GuRoBi solver package in AMPL for solving the QC-MINLP model is appropriate given its capability to handle such complex models. This is consistent with the methodologies adopted by [24] and others in similar optimization challenges.

The use of mixed-integer non-linear programming (MINLP) models is well documented in the literature for optimizing complex logistics and transportation problems. For instance, [13] utilized a robust fuzzy mathematical programming model to address uncertainties in supply chain network design, demonstrating the feasibility of such advanced mathematical models for real-world applications. The manuscript's QCMINLP model aligns with existing approaches that employ complex mathematical models to address transportation and logistics challenges, particularly in scenarios involving interconnected variables.

### 3.4 Data collection

Numerous data points were used in this study and collected from different sources. This is a case-based study, and most of the parameters were collected from the factory. Data regarding traffic and distance were gathered using GPS and Google Maps. Table 3 show the parameters and their sources.

**Table 3:** Parameters and their sources.

Parameters	Sources
types of vehicles, vehicle space, cargo weight, fuel consumption rates, maintenance costs	factory vehicle pool unit
volume, weight, and package dimensions of products	factory production unit
route distances, travel times	GPS data, historical traffic patterns, Google Map
demand	historical sales data

Some uncertainties in the data were analyzed. Fluctuations in demand at different retailers can affect delivery schedules. Unplanned maintenance or breakdowns can disrupt schedules. These uncertainties were addressed through robust data collection and analysis. The proposed model aims to provide reliable and efficient optimization for transportation systems in supply chains.

## 4 Result and discussion

**Figure 3:**
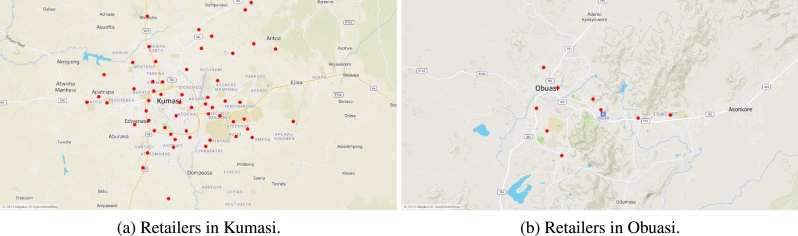
Location of some retailers in Kumasi and Obuasi.

**Figure 4:**
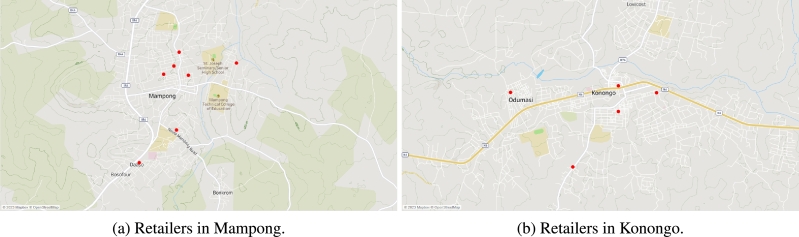
Location of some retailers in the Mampong and Konongo.

Fig. 3a shows the location of retailers in Kumasi. Fig. 3b shows the location of the nine retailers in Obuasi. Fig. 4a shows the location of the seven retailers in Mampong. Fig. 4b shows the location of the five retailers in Konongo. There are a total of 77 retailers (RT1,⋯,RT77) scattered across these four communities. The warehouses transport four finished products: drum, sachet_1_, sachet_2_, sachet_3_, and sachet_4_, and have three types of vehicles: truck_1_, truck_2_, and a van in its transportation fleet.

**Summary**

The formulated model took into account various factors, including delivery schedules, working days, and time slots for all days. The solution to the optimization model above was found in 6 hours and 7 minutes. The model has 2,133,120 decision variables. Again, there are 682,416 constraints, of which 658,350 are non-linear and 24,066 are linear. Additionally, there are 408 equality constraints and 682,008 inequality constraints.

**Figure 5:**
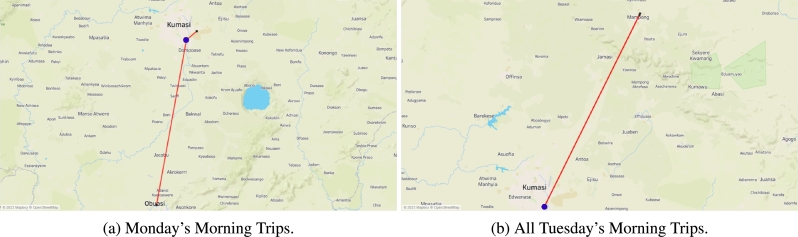
Single trips made in the distribution phase.

**Figure 6:**
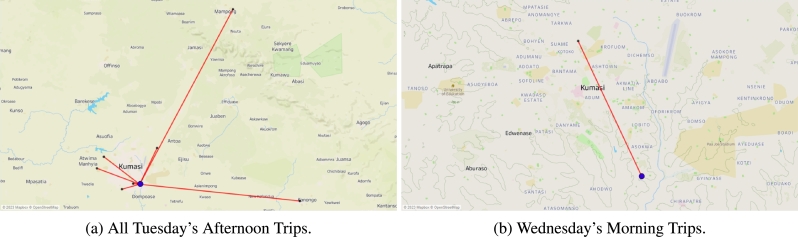
Multiple and single trips.

The model involved transporting a forecasted demand from the factory to the warehouse, which required the use of a single 40-mt vehicle for long-distance transport (single sink). The distribution process started on a Monday morning, as illustrated in Fig. 5a, but only two retailers (RT35 and RT38) were served due to heavy traffic. This involved using two separate vehicles, a truck and a van, to transport products to these retailers, with one located in Obuasi and the other in Kumasi. Moving on to Tuesday morning, two vehicles were dispatched for deliveries to retailers RT76 and RT77, as depicted in Fig. 5b. However, it's worth noting that on Tuesday afternoon, the distribution operations were quite fragmented, as shown in Fig. 6a.

**Figure 7:**
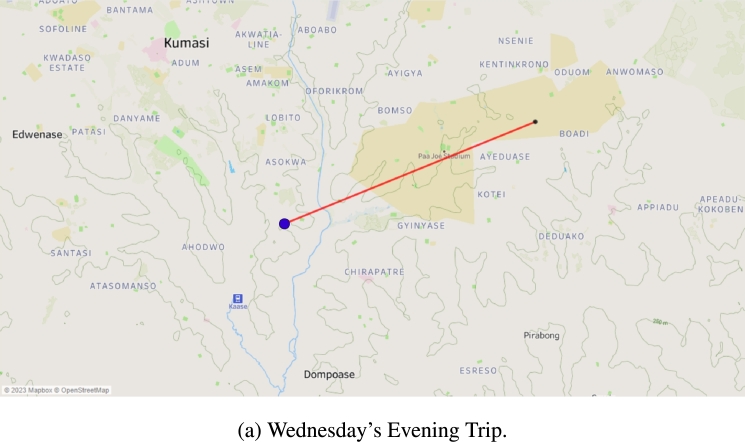
Single trips made in the distribution phase.

Instead of a continuous flow of deliveries, multiple separate distribution trips occurred, covering retailers RT24, RT33, RT63, RT67, RT68, RT69, and RT71. On Wednesday morning, there was a single trip from the warehouse to RT26, as indicated in Fig. 6b, with the use of a single van for distribution. Similarly, on Wednesday evening, there was a single trip to RT8, showcased in Fig. 7a, with the same single van for transportation.

In Fig. 7, deliveries were made individually because of the diverse and dispersed locations of the retailers, making it challenging to consolidate them into a single trip efficiently.

In such cases, individual trips were the most optimal approach.

**Figure 8:**
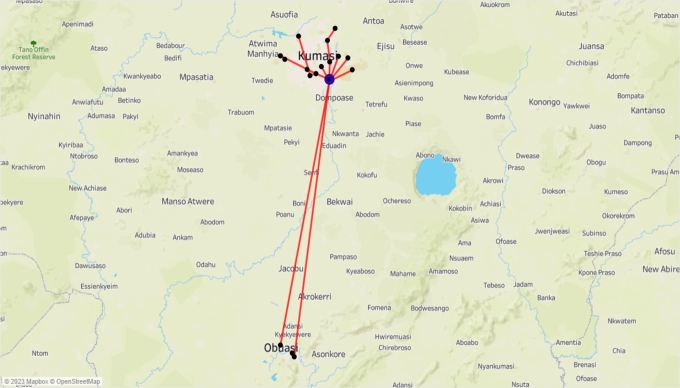
All Wednesday's afternoon trips.

On Wednesday afternoon, a total of 17 retailers were visited, as shown in Fig. 8, in a continuous schedule, visiting one retailer after the other.

**Figure 9:**
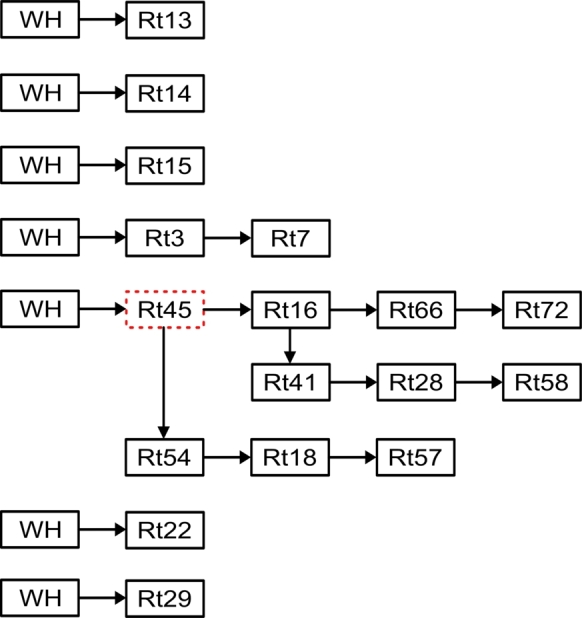
Wednesday's afternoon delivery sequence.

As seen from Fig. 9, RT13, RT14, RT15, RT3, RT45, RT22, and RT29 were directly visited from the warehouse, and all other retailers were visited via other retailers, forming a chain of deliveries from one retailer to the next.

On Wednesday afternoon, multiple continuous delivery sequences occurred, involving the use of both single and multiple vehicles. In the initial sequence, goods were transported to RT13. The second sequence involved the delivery of goods to RT14, while in the third sequence, goods were transported to RT15. In the fourth sequence, goods were transported to both RT3 and RT7. The fifth sequence employed three vehicles used for transporting goods. Two of these vehicles traveled from the warehouse through RT45 to subsequently deliver goods to RT16, RT66, and RT72. At RT16, one of the vehicles diverted to make additional deliveries to RT41, RT28, and RT58. The third vehicle also passed through RT45 from the warehouse and delivered goods to RT54, RT18, and RT57.

It's worth noting that in the fifth sequence, the delivery process began at the warehouse and proceeded through RT45 and RT16 before experiencing a route diversion. This implies that the initial route for both parts of the sequence included a passage through RT16. It's important to clarify that passing through a retailer's location, like RT45, doesn't necessarily mean a delivery has been made there; it may simply be the most efficient route. To distinguish between locations where deliveries are made and those where deliveries aren't made when a route is used, we can observe the quantity of goods in the vehicle. A reduction in cargo indicates deliveries have been made at such locations. Finally, the sixth and seventh sequences involved the delivery of goods to RT22 and RT29, respectively.

Let's look at the distribution from WH to RT45, RT54, RT18, and RT57 in detail.

As indicated in Table 5, the following quantities of products were loaded into the single truck: 19 boxes of Drum, 21 boxes of Sachet_1_, 28 boxes of Sachet_2_, 23 boxes of Sachet_3_, and 27 boxes of Sachet_4_. Again, in two of the vans (A and B), the following quantities of products were loaded: 33 boxes of Drum, 36 boxes of Sachet_1_, 36 boxes of Sachet_2_, 35 boxes of Sachet_3_, and 37 boxes of Sachet_4_. This marks the initial trip from the warehouse (WH1) to the retailer (RT45), and all three vehicles were in transit from the warehouse to RT45.

Retailer 45 (RT45) requires the following quantities: 9 boxes of Drum, 5 boxes of Sachet_1_, 10 boxes of Sachet_2_, 9 boxes of Sachet_3_, and 7 boxes of Sachet_4_, as shown in Table 4.

**Table 4:** Demand for RT54, RT18, and RT57.

Retailers	Drum	Sachet_1_	Sachet_2_	Sachet_3_	Sachet_4_
RT54	10	9	10	5	6
RT18	10	7	0	6	5
RT57	0	5	5	10	8
**Total**	20	21	15	21	19

**Table 5:** AMPL solution snippet of warehouse to RT45.

Day	Origin	Destination	Time	Vehicle	Product	Quantity
Wed	WH1	RT45	Afternoon	Truck_2_	Drum	19
Wed	WH1	RT45	Afternoon	Truck_2_	Sachet_1_	21
Wed	WH1	RT45	Afternoon	Truck_2_	Sachet_2_	28
Wed	WH1	RT45	Afternoon	Truck_2_	Sachet_3_	23
Wed	WH1	RT45	Afternoon	Truck_2_	Sachet_4_	27

Wed	WH1	RT45	Afternoon	Van_A+B_	Drum	33
Wed	WH1	RT45	Afternoon	Van_A+B_	Sachet_1_	36
Wed	WH1	RT45	Afternoon	Van_A+B_	Sachet_2_	36
Wed	WH1	RT45	Afternoon	Van_A+B_	Sachet_3_	35
Wed	WH1	RT45	Afternoon	Van_A+B_	Sachet_4_	37

Table 6 further illustrates the goods carried by each of the three vehicles.

**Table 6:** AMPL solution snippet of RT45 to RT16 and RT54.

Day	Origin	Destination	Time	Vehicle	Product	Quantity
Wed	RT45	RT16	Afternoon	Truck_2_	Drum	19
Wed	RT45	RT16	Afternoon	Truck_2_	Sachet_1_	16
Wed	RT45	RT16	Afternoon	Truck_2_	Sachet_2_	18
Wed	RT45	RT16	Afternoon	Truck_2_	Sachet_3_	23
Wed	RT45	RT16	Afternoon	Truck_2_	Sachet_4_	27

Wed	RT45	RT16	Afternoon	Van_A_	Drum	13
Wed	RT45	RT16	Afternoon	Van_A_	Sachet_1_	15
Wed	RT45	RT16	Afternoon	Van_A_	Sachet_2_	21
Wed	RT45	RT16	Afternoon	Van_A_	Sachet_3_	19
Wed	RT45	RT16	Afternoon	Van_A_	Sachet_4_	11

Wed	RT45	RT54	Afternoon	Van_B_	Drum	20
Wed	RT45	RT54	Afternoon	Van_B_	Sachet_1_	21
Wed	RT45	RT54	Afternoon	Van_B_	Sachet_2_	15
Wed	RT45	RT54	Afternoon	Van_B_	Sachet_3_	16
Wed	RT45	RT54	Afternoon	Van_B_	Sachet_4_	19

As observed in Table 6, the contents of the truck were reduced for Sachet_1_, from 21 to 16. Notably, the difference in quantity matches precisely with the quantity of Sachet_1_ demanded by RT45. This same reduction was made for Sachet_2_ to match with RT45's demand. Additionally, the vans have experienced a reduction of 7 boxes of Sachet_4_, which were offloaded for RT45. At this stage, RT45 has received a portion of its demand, which includes Sachet_1_, Sachet_2_, and Sachet_4_. The remaining items, Drum and Sachet_3_, will be delivered in the upcoming days within the week. At this stage, one truck and a van_A_ have been dispatched to RT16 from RT45, while another van_B_ has been assigned to move to RT54 from RT45. Table 7 shows the van arriving at RT54 from RT45 and transiting to RT18.

**Table 7:** AMPL solution snippet of RT54 to RT18.

Day	Origin	Destination	Time	Vehicle	Product	Quantity
Wed	RT54	RT18	Afternoon	Van_B_	Drum	10
Wed	RT54	RT18	Afternoon	Van_B_	Sachet_1_	12
Wed	RT54	RT18	Afternoon	Van_B_	Sachet_2_	5
Wed	RT54	RT18	Afternoon	Van_B_	Sachet_3_	16
Wed	RT54	RT18	Afternoon	Van_B_	Sachet_4_	13

Retailer 54 (RT54) also requires the following quantities: 10 boxes of Drum, 9 boxes of Sachet_1_, 10 boxes of Sachet_2_, 5 boxes of Sachet_3_, and 6 boxes of Sachet_4_, as shown in Table 4. Except for Sachet_3_, all the required quantities of products for RT54 were offloaded before the van was redirected to RT18. Table 7 shows the van arriving at RT18 from RT54 and transiting to RT57. Again, Retailer 18 (RT18) requires the following quantities: 10 boxes of Drum, 7 boxes of Sachet_1_, 6 boxes of Sachet_3_, and 5 boxes of Sachet_4_.

All the required quantities for RT18 were offloaded before the van carried the needed quantities, including 5 boxes of Sachet_1_, 5 boxes of Sachet_2_, 10 boxes of Sachet_3_, and 8 boxes of Sachet_4_, to RT57, effectively concluding the distribution process as shown in Table 8. As evident from Table 7, the quantity sent from RT18 to RT57 matched the required quantity as shown in Table 4. It is worth noting that RT57 did not require Drum, and the delivery was structured to ensure that all Drum products were offloaded before reaching RT57. The summary is shown in Table 9 where “0” indicate the retailer does not require that product and “-” indicate the retailer requires the product but it wasn't delivered by that vehicle or in that shipment.

**Table 8:** AMPL solution snippet of RT18 to RT57.

Day	Origin	Destination	Time	Vehicle	Product	Quantity
Wed	RT18	RT57	Afternoon	Van_B_	Sachet_1_	5
Wed	RT18	RT57	Afternoon	Van_B_	Sachet_2_	5
Wed	RT18	RT57	Afternoon	Van_B_	Sachet_3_	10
Wed	RT18	RT57	Afternoon	Van_B_	Sachet_4_	8

**Table 9:** Summary of goods distributed by a van from warehouse to RT57.

Product	RT45	RT54	RT18	RT57	**Total**
Drum	-	10	10	0	20
Sachet_1_	-	9	7	5	21
Sachet_2_	-	10	0	5	15
Sachet_3_	-	-	6	10	16
Sachet_4_	-	6	5	8	19
**Total**	-	35	28	28	

**Figure 10:**
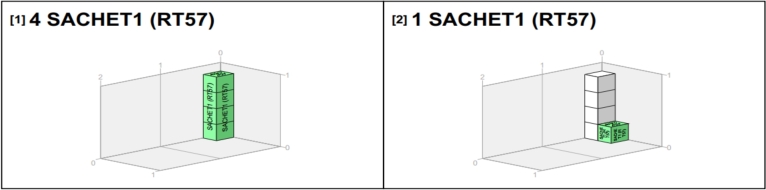
Packing arrangement for Sachet_1_.

**Figure 11:**
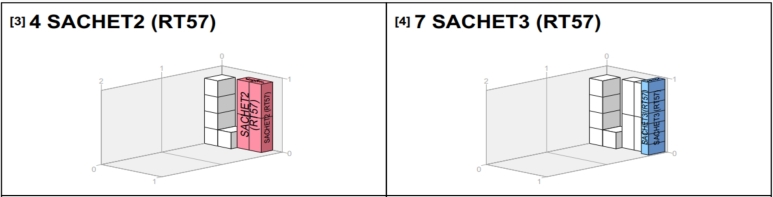
Packing arrangement for Sachet_2_.

**Figure 12:**
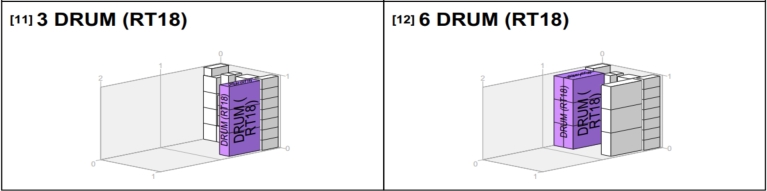
Packing arrangement for Drum.

**Figure 13:**
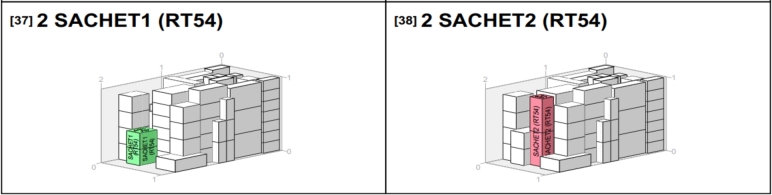
Packing arrangement for Sachet_1_ and Sachet_2_.

Since the goods from the van_B_ weren't all delivered to a single location, but rather 35, comprising different products at RT54, 28 at RT18, and 28 at RT57, it's important to determine the best way to pack them for efficient offloading. This study used the PackVol container loading optimization software alongside the models we've created to make better decisions. The model helped plan transportation routes, choose the right vehicles, and determine how much of each product should go in each vehicle. PackVol, on the other hand, helped efficiently pack the products, considering the order in which they needed to be delivered. Figure 10, Figure 11, Figure 12, Figure 13 shows the guidelines products.

As shown in Fig. 10, the packing arrangement for Sachet_1_ in the van destined for RT57 is organized in such a way that Sachet_1_ and all other products needed by RT57 were loaded first since RT57 was the last retailer to be visited. This ensures that the items required by RT57 do not interrupt the delivery process of other retailers who will be delivered before RT57.

As illustrated in Fig. 11, the packing arrangement for Sachet_2_ and Sachet_3_ in the van destined for RT57 is shown. It's worth noting that, based on the information in Table 9, RT57 does not require Drum. Therefore, even though other retailers may require Drum, it is not added at this stage as it's not needed for RT57. This ensures that the packing is tailored specifically to meet the requirements of RT57.

In Fig. 12, you can see how we've packed Drum products specifically for RT18. This is because RT18 requires these Drum products, so it's important to include them in the packing arrangement.

In Fig. 13, you can observe how we've organized the packing of products for the initial retailer in the delivery sequence, which happens to be RT54. Since this retailer is the first stop on the route, its goods are packed last, ensuring they are positioned at the rear of the van for easy access when making the first delivery.

**Figure 14:**
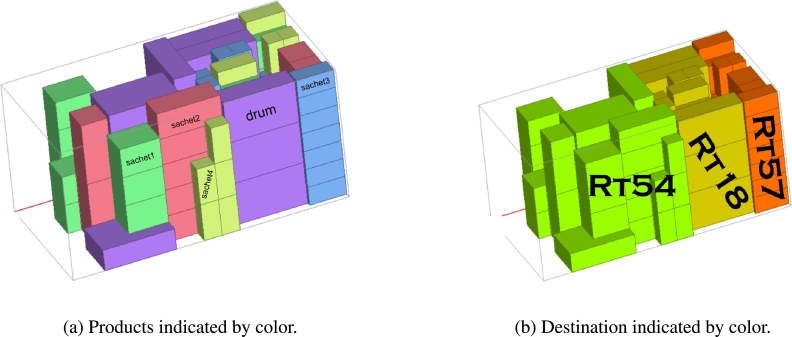
Product categorized by products and destinations.

In Fig. 14, you can see how we've packed the goods to make the unloading process smoother, keeping in mind the order in which the retailers will be visited.

In Fig. 14a, each product has its own unique color, while in Fig. 14b, each retailer is represented by a specific color. When we compare these two images, it becomes clear that the contents being transported, or the loads, are identical in both cases. The majority of the trips occurred on Saturday, spanning from morning to afternoon and into the evening.

**Figure 15:**
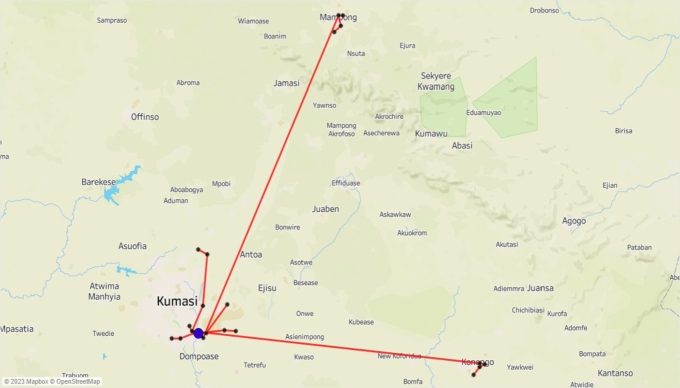
All Saturday's morning trips.

Fig. 15 shows all the trips for Saturday morning.

**Figure 16:**
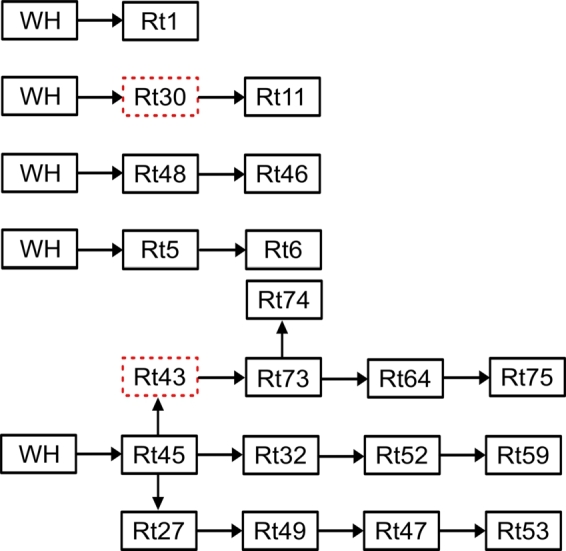
Saturday's morning delivery sequence.

As seen from Fig. 16, RT1, RT48, RT5, RT11, and RT45 were directly visited from the warehouse, and all other retailers were visited via other retailers, forming a chain of deliveries from one retailer to the next.

**Figure 17:**
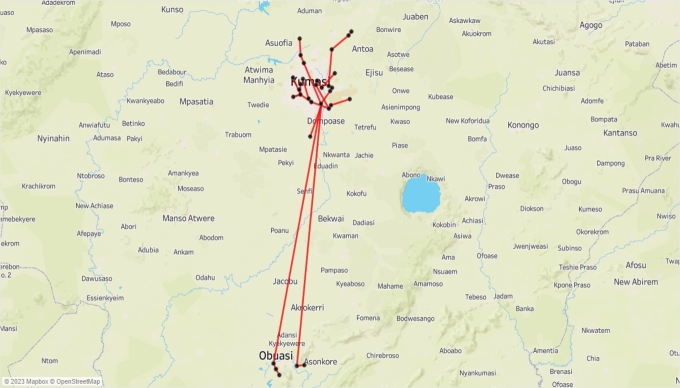
All Saturday's afternoon trips.

In the first sequence in Fig. 16, goods were transported from the warehouse to RT1. In the second sequence, goods were transported from the warehouse, passed through RT30 without delivering, and then proceeded to RT11 for the final delivery. The third sequence involved transporting goods from the warehouse to RT48 and subsequently to RT46. In the fourth sequence, goods were transported from the warehouse to RT5 and then to RT6. In the fifth sequence, goods were transported from the warehouse to RT45 using four vehicles. Some goods were unloaded at RT45, while two vehicles continued to RT43, not unloading there but using this route to deliver goods to RT73, RT74, RT64, and RT75. One of the four vehicles that left the warehouse via RT45 was headed to deliver goods to RT32, RT52, and RT59. Finally, the last vehicle also traveled through RT45 to deliver goods to RT27, RT49, RT47, and RT53 (Fig. 17).

**Figure 18:**
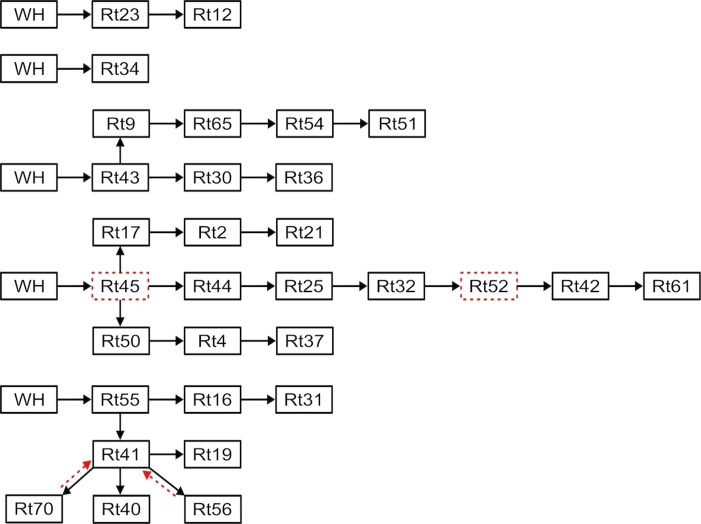
Saturday's afternoon delivery sequence.

As seen in Fig. 18, RT23, RT34, RT43, RT17, RT50, and RT55 were directly visited from the warehouse, and all other retailers visited via other retailers, forming a chain of deliveries from one retailer to the next.

In the first sequence in Fig. 18, goods were transported from the warehouse to RT23 and RT12. In the second sequence, goods were transported exclusively to RT34. In the third sequence, two vehicles transported goods from the warehouse to RT43. After delivering the goods at RT43, one of the vehicles proceeded to deliver goods to RT9, RT65, RT54, and RT51. The other vehicle continued from RT43 to deliver goods to RT30 and RT36. In the fourth sequence, three vehicles were used. The first vehicle delivered goods to RT17, RT2, and RT21, passing through RT45. The second vehicle also delivered goods to RT44, RT25, RT32, RT42, and RT61, passing through RT45 and RT52. The last vehicle delivered goods to RT50, RT4, and RT37, passing through RT45. In the final sequence, three vehicles were deployed. The first vehicle delivered goods to RT55, RT16, and RT31. The other two vehicles initially moved to RT41, with one vehicle then proceeding to RT19 for its final delivery. The remaining vehicle, which initially went to RT41, continued on to RT40, then returned to RT41, followed by stops at RT56, and ultimately, after multiple stops at RT41, reached its final destination at RT70. The total transportation cost computed by Equation (1) is estimated at GH¢20,270 ($1,638.91) when the fuel price was GH¢16 ($1.29).

In examining the distribution process outlined in the results discussion, it becomes clear that the distribution of the products in long-hauling and intercity scenarios is a complex and dynamic operation, especially in the latter. One of the critical aspects of the distribution process conducted in this study is the importance of adaptability and resource efficiency. The study highlights how traffic conditions and retailer locations necessitate flexible and adaptive strategies. This emphasizes the need for distribution systems to be agile and capable of responding to real-time challenges, such as traffic congestion, to ensure efficient resource utilization.

In current research, authors have made similar findings about the sustainability of freight transportation [5]. This study elaborates on the significance of advanced route planning and optimization tools to minimize travel time, reduce fuel consumption, and enhance overall cost-effectiveness. The use of various vehicles, including trucks and vans, reflects the practicality of a multi-modal distribution approach. This model significantly reduces transportation costs by 27.59%, from GH¢20,270 ($1,638.91) to GH¢14,676 ($1,186.61) on average, showcasing its effectiveness in reducing the logistic operational cost for the industry.

Our results also show the merits of creating a chain of deliveries to reach multiple retailers efficiently, which is a valuable strategy. Again, our results show that the efficient packaging of goods in vehicles is crucial for both maximizing cargo capacity and facilitating unloading. The model has provided substantial reductions in fuel, operational, and maintenance costs for the company, which will improve delivery schedules and route optimization. The model have also provided faster and more reliable delivery of goods, ensuring effective delivery operations. A similar finding was made in green logistics management and supply chain system construction based on internet of things technology [21]. Our results also show that Saturdays are the ideal day for distribution, as already indicated in a current study by [25], stating that better urban traffic conditions are on Saturdays and Sundays.

### 4.1 Post-optimal analysis

The post-optimal analysis helps evaluate the robustness and sensitivity of the obtained results against key parameters in the model. We look at two key parameters: fuel price (FP), and vehicle capacity (VC).

The sensitivity analysis presented in the Table 10 examines the impact of changes in fuel prices on transportation costs. The analysis shows that a 10% decrease in fuel prices results in a 1.22 percent decrease in transportation costs, while a 10% increase in fuel prices results in a 1.22 percent increase in transportation costs. This shows a roughly symmetrical impact of fuel price changes on transportation costs.

**Table 10:** Fuel Price Sensitivity.

Scenario	Fuel Price Change	Transportation Cost	Cost Change
Baseline	0%	$1,186.61	0%
Scenario 1	-10%	$1,172.07	(-)1.22%
Scenario 2	+10%	$1,201.15	(+)1.22%
Scenario 3	+20%	$1,215.69	(+)2.45%

From Table 11, we noticed that a 10% decrease in vehicle capacity leads to a significant 19.88% increase in transportation costs. This indicates that reducing vehicle capacity has a negative impact on transportation costs. Increasing vehicle capacity by 10% to 20% results in a 5.30% decrease in transportation costs, while a 21% to 30% increase in capacity leads to a 9.55% decrease in costs. This shows that increasing vehicle capacity can lead to considerable cost savings, though the impact is less pronounced than the cost increase from decreased capacity.

**Table 11:** Vehicle Capacity Sensitivity.

Scenario	Vehicle Capacity Change	Transportation Cost	Cost Change
Baseline	0%	$1,186.61	0%
Scenario 1	-10%	$1,422.51	(+)19.88%
Scenario 2	+10% - 20%	$1,123.67	(-)5.30%
Scenario 3	+21% - 30%	$1,073.22	(-)9.55%

## 5 Conclusion

In conclusion, our examination of an effective approach to distributing processed agricultural products via long-hauling and intercity distribution reveals key benefits that align with the real-world distribution scenarios described in our discussion. Our analysis has shown that adapting distribution strategies in response to various factors such as traffic conditions, retailer locations, and specific needs is crucial for achieving efficiency. By optimizing vehicle assignments, routes, and shipment activation, our model mirrors the observed approach of using different vehicles and routes to meet distribution goals. Just as observed, our model highlights the significance of using multiple vehicles when needed to efficiently deliver to multiple retailers simultaneously. Efficient resource utilization, as seen in the discussion, is a central objective of our model. It aims to minimize operational costs while meeting the demands of various retailers. The model also addresses the challenges of efficient packing and unloading by considering cargo capacity and product arrangement within vehicles. Our model highlights the role of data-driven decision-making, utilizing mathematical optimization techniques to determine the most cost-effective distribution strategies.

The utilization of our model is expected to significantly cut down transportation costs, currently at an estimated cost of GH¢14,676 ($1,186.61). The research hypothesis tends to be confirmed, as results indicate a substantial 27.59% cost reduction, which highlights how well our strategy works in lowering fuel, operational, and maintenance costs while making sure the delivery of tomato paste to retailers is smooth and efficient.

By reducing transportation costs, the company can offer more competitive pricing, thus gaining a market edge over competitors. The model supports sustainability initiatives by optimizing routes to reduce fuel consumption and emissions, aligning with corporate social responsibility goals. Incorporating real-time data and robust optimization techniques allows managers to better handle uncertainties, enhancing supply chain resilience. Again, optimizing vehicle usage ensures that resources are allocated efficiently, minimizing waste and maximizing productivity. Lastly, improved delivery reliability and timeliness can enhance customer satisfaction and loyalty, contributing to long-term business success.

These are some of the limitations posed by the method used to solve this problem. The model's accuracy relies heavily on the quality and availability of real-time data, and inaccurate or incomplete data can lead to suboptimal results. Again, while effective for medium-sized networks, the model's computational complexity may increase significantly for larger-scale applications, potentially limiting its scalability. Our model assumes relatively stable demand, which may not reflect real-world fluctuations. It is recommended to consider more dynamic demand scenarios. Again, future studies can apply the model to different commodities to evaluate its versatility and adaptability in other markets, not just tomato pastes.

## CRediT authorship contribution statement

**Bernard Atta Adjei:** Writing – review & editing, Writing – original draft, Validation, Software, Methodology, Conceptualization. **Charles Sebil:** Supervision, Formal analysis. **Dominic Otoo:** Writing – original draft, Supervision. **Joseph Ackora-Prah:** Supervision, Methodology, Investigation.

## Declaration of Competing Interest

The authors declare that they have no known competing financial interests or personal relationships that could have appeared to influence the work reported in this paper.

## Data Availability

The data supporting the findings of this study are available upon request. Please contact the corresponding author for access to the data.

## References

[1] Abdul S., Khan R., Zhang Y. (2017). The cost minimization model in warehouse distribution system. Int. J. Manag. Fuzzy Syst..

[2] Angueiraa J., Konduria K., Chakourb V., Eluruc N. (2017). Exploring the relationship between vehicle type choice and distance travelled: a latent segmentation approach. Int. J. Transp. Res..

[3] Appiah S.T., Otoo D., Adjei B.A. (2020). A multi-vehicle, multi-factory assignment problem: a case of coca-cola bottling company at Ahinsan and Spintex-Ghana. Am. J. Oper. Res..

[4] Bevrani B., Burdett R.L., Bhaskar A., Yarlagadda P.K. (2017). A capacity assessment approach for multi-modal transportation systems. Eur. J. Oper. Res..

[5] Browne M., Dubois A., Hulthén K. (2023). Transportation as a loosely coupled system: a fundamental challenge for sustainable freight transportation. Int. J. Sustain. Transp..

[6] Branthome F.X. (2021).

[7] Ceder A. (2011). Optimal multi-vehicle type transit timetabling and vehicle scheduling. Proc., Soc. Behav. Sci..

[8] Chen P., Golden B., Wang X., Wasil E. (2017). A novel approach to solve the split delivery vehicle routing problem. Int. Trans. Oper. Res..

[9] Davda P., Patel J. (2019). Developed method for optimal solution of transportation problem. Int. J. Res. Eng. Technol..

[10] Faragó I., Georgiev K., Havasi Á., Zlatev Z. (2014). Efficient algorithms for large scale scientific computations: introduction. Comput. Math. Appl..

[11] Farmand N., Zarei H., Rasti-Barzoki M. (2021). Two meta-heuristic algorithms for optimizing a multi-objective supply chain scheduling problem in an identical parallel machines environment. Int. J. Ind. Eng. Comput..

[12] Ghaffari Z., Nasiri M.M., Bozorgi-Amiri A., Rahbari A. (2020). Emergency supply chain scheduling problem with multiple resources in disaster relief operations. Transportmetrica A: Transp. Sci..

[13] Ghahremani-Nahr J., Kian R., Sabet E. (2019). A robust fuzzy mathematical programming model for the closed-loop supply chain network design and a whale optimization solution algorithm. Expert Syst. Appl..

[14] Golden B.L., Raghavan S., Wasil E.A. (2008).

[15] Holzapfel A., Potoczki T., Kuhn H. (2023). Designing the breadth and depth of distribution networks in the retail trade. Int. J. Prod. Econ..

[16] James J., Yu W., Gu J. (2019). Online vehicle routing with neural combinatorial optimization and deep reinforcement learning. IEEE Trans. Intell. Transp. Syst..

[17] Jeter M.W. (2018).

[18] Jungwirth A., Frey M., Kolisch R. (2020).

[19] Leite M., Santos S.C., Junior W.R.G. (2015). Transportation modal choice in coolant importation through total cost minimization: a case study. Ind. J. Manag. Prod..

[20] Litman T. (2017).

[21] Liu C., Ma T. (2022). Green logistics management and supply chain system construction based on internet of things technology. Sustain. Comput. Inf. Syst..

[22] Manuel O., Alexander H. (2018). Vehicle selection for a multi-compartment vehicle routing problem. Eur. J. Oper. Res..

[23] McCann P. (2001). A proof of the relationship between optimal vehicle size, haulage length and the structure of distance-transport costs. Transp. Res., Part A, Policy Pract..

[24] Muley V.Y. (2021). Modeling Transcriptional Regulation.

[25] Muñoz-Villamizar A., Solano-Charris E., AzadDisfany M., Reyes-Rubiano L. (2021). Study of urban-traffic congestion based on Google maps API: the case of Boston. IFAC-PapersOnLine.

[26] Onwude D.I., Chen G., Eke-Emezie N., Kabutey A., Khaled A.Y., Sturm B. (2020). Recent advances in reducing food losses in the supply chain of fresh agricultural produce. Processes.

[27] Phiboonbanakit T., Horanont T., Huynh V.N., Supnithi T. (2021). A hybrid reinforcement learning-based model for the vehicle routing problem in transportation logistics. IEEE Access.

[28] Ramesh G., Sudha G., Ganesan K. (2018). Solution of two vehicle cost varying interval transportation problem-a new approach. Int. J. Pure Appl. Math..

[29] Ríos-Mercado R.Z., Borraz-Sánchez C. (2015). Optimization problems in natural gas transportation systems: a state-of-the-art review. Appl. Energy.

[30] Song L., Wu Z. (2023). An integrated approach for optimizing location-inventory and location-inventory-routing problem for perishable products. Int. J. Transp. Sci. Technol..

[31] Wu W., Ma J., Liu R., Jin W. (2022). Multi-class hazmat distribution network design with inventory and superimposed risks. Transp. Res., Part E, Logist. Transp. Rev..

[32] Wu W., Zhou W., Lin Y., Xie Y., Jin W. (2021). A hybrid metaheuristic algorithm for location inventory routing problem with time windows and fuel consumption. Expert Syst. Appl..

[33] Yin Y., Wang J., Chu F., Wang D. (2024). Distributionally robust multi-period humanitarian relief network design integrating facility location, supply inventory and allocation, and evacuation planning. Int. J. Prod. Res..

[34] Zhou Y., Wang J., Yang H. (2019). Resilience of transportation systems: concepts and comprehensive review. IEEE Trans. Intell. Transp. Syst..

